# Decomposition of Tinct. Opii by Ammonia

**Published:** 1845-01

**Authors:** 


*Decomposition of Tinct. Opii by Ammonia.*—It is of great importance for prescribers to remember that the addition of ammonia, either as carbonate or spiritus ammon. aromaticus, to mixtures containing tincture of opium or any salt of morphia, will, after some time, say twenty-four hours, precipitate the morphia in a crystalline form; so that if a mixture is made a day or two before it is taken, the patient may get several doses of morphia concentrated in the last portion left in the bottle, and fatal consequences may be produced. The presence of alcohol will prevent the precipitation.

*Chemical Gazette.*

